# The Use of Instruments in Obstetric Practice

**Published:** 1860-12

**Authors:** 


### THE USE OF INSTUMENTS IN OBSTETRIC PRACTICE.

The use and abuse of instruments in midwifery is a theme worthy of strict examination, and hitherto we believe much misapprehension has existed on the subject. In the schools and in systematic works, it is laid down as a rule, that the use of instruments is never to be had recouse to if it can possibly be avoided, and young men generally commence practice under the direst fear and apprehension of committing errors in this respect. Indeed, so strictly are they cautioned against the use of instruments in midwifery, that they scarcely think it worth while to learn how to manage them; consequently, when they meet with a case requiring instrumental aid, they feel they have a great emergency to contend with, without the necessary qualification to meet it; and many a valuable life may have been placed in jeopardy, or lost owing to the indecision or incompetency of an untaught junior practitioner. Surely it would be a much safer method of teaching to make students experienced in the use of instruments, leaving it afterwards to their own judgment and sense of propriety not to abuse them, than to instil into their minds such a horror of them, t hat when thy do meet with difficulties in practice and have to depend upon their own resources, they either shirk necessary instrumental aid or, if driven to employ instruments, find themselves baffled, to the peril of the lives of those entrusted to their care. Dr. Tyler Smith strongly urges the more frequent employment of the forceps as one of the means of removing the necessity for craniotomy, and remarks that "there is scarely any point in practice admitting of greater improvement than the more frequent use of this instrument in the capacity of extractor, of lever, of rotator, of compressor, and lastly, of an excitor of uterine action." He states, that "the maternal mortality, after the use of the forceps, is in this country somewhat less than one in twenty, whereas nearly one in five of all mothers die after craniotomy." Now taken into calculation what a comparatively easy operation craniotomy is, the greater mortality attending it probably *depends more upon the consequences of pressure and exhaustion from protracted labor, than upon the operation itself*, and is a strong argument in favor of the use of the forceps at an earlier period. But the argument applies with still greater force in favor of the vectis, which can not only be used earlier in labor than the forceps, but is followed by a much smaller rate of maternal mortality, as well as of certain other unfavorable results of delivery by the forceps. The writer of this article used the vectis in private practice one hundred and twenty times, and not one of the mothers died. He has since used it in *consultation* practice, frequently after failure of the forceps in experienced hands, 56 times in 210 labors, or 1 in 3-75, with a fatal result to one mother only, and that from puerperal fever after a very prolonged labor. Scarcely ever has it caused rupture of the perineum, and in no instance sloughing or injurious iflnammation of the soft parts. How is it that an instrument of such value is scarcely mentioned in systematic works, and not at all by the author of the paper in question? We know that it is very extensively used in provincial midwifery practice, and depended upon by many to the exclusion of the forceps; Denman, a great authority, thought highly of it, and the very argument used by those who inculcate in the minds of students the axiom that "meddlesome midwifery is bad"—viz., that practitioners are too apt to use them more frequently than necessary, implies in some degree that benefit is derived from their employment; for, undoubtedly, no practitioner would frequently resort to any means of treatment which he found predjudicial to his patients, and consequently injurious to his own reputation and success. In making these remarks, however, we are anxious not to be misunderstood; we deprecate and interference with the natural process of labor when all is going on well; we disallow any excuse for instrumental interference on the score of convenience or saving of time to the practitioner; but we nevertheless agree with the opinion of Dr. Tyler Smith, that "*greater dangers accrue from the neglect of instrumental interference than from the abuse of these means*, and that the evils which result from protracted labors are now so well understood, that few obstetricians could be found who would seriously advocate the barbarous practice of keeping a woman undelivered for several hours after her delivery had become safe and practicable by the forceps."

There is another very interesting paper in the "Transactions," on "The more frequent Use of the Forceps as a means of lessening both Maternal and Fœtal Mortality, by P. H. Harper. who pleads strongly, both by argument and statistics, in favor of a much more frequent employment of the instrument. He has endeavored to show there is no special maternal risk which necessarily attaches to the use of forceps, and that the various injuries and other ill effects which are usually attributed to them are not the result of their use but of their abuse. That when maternal death occurs after their use, it is generally because of the result of the length of time the labor has lasted before their application. That so long as they are applied as the last resort in tedious labor, so long will the maternal mortality after their use appear to be very high.

"We have proved," he says, "from the only statistics which give us the necessary data. where the forceps were freely used, that 1 mother in 22, and 1 child in 5 died in unassisted tedious labor; whilst 1 mother in 56, and 1 child in 84 died where the forceps were used; and 1 mother in 10 died after craniotomy. This proves that the maternal death is less after the use of the forceps than after craniotomy, or even unassisted tedious labor."

We subjoin the following table, which tells its own tale:

| | Forceps cases. | Fœtal deaths. | Maternal deaths. | Duration. |
|---|---|---|---|---|
| Collins.... | 1 in 694.... | 1 in 26.... | 1 in 329.... | 38 hours. |
| Hardy..... | 1 in 355.... | 1 in 20.... | 1 in 334.... | 35½ " |
| Johnston .. | 1 in 60.... | 1 in 35.... | 1 in 502.... | 29½ " |
| Harper.... | 1 in 26.... | 1 in 47.... | 1 in 1490.... | 16 " |

—*Brit. and For. Medico-Chirurgical Review.*

Carelessness on the part of Correspondents.—The Journal of Materia Medica complains of the inconvenience caused by the neglect of letter writers to give their addresses in full with post office, county, State, &c. We presume that every one having an extensive correspondence is frequently annoyed in the same way. We are sometimes unable to reply to letters, either from inability to decipher the name of the writer, or for lack of the county and State being stated. There is a great need of reform in this particular. Subscribers, too, who change their location, and wish the address of their Journal changed, also, should in all cases state the post office to which it is at present sent, as well as the alteration they wish effected.

Removal of the Tongue.—Dr. Choppin reports, in the N. O. *Medical News*, the successful removal of the tongue in a case of cancer, with the ecraseur. The operation lasted fifteen minutes, and was performed without hemorrhage. This is one of the kind of cases to which the ecraseur is admirably adapted.

